# Supplementary material for: Moral conformity in a digital world: Human and nonhuman agents as a source of social pressure for judgments of moral character
Source: PLoS One. 2024 Feb 15;19(2):e0298293. doi: 10.1371/journal.pone.0298293 (PMC10868870; doi:10.1371/journal.pone.0298293)
Supplement: S1 File — (DOCX) [file pone.0298293.s001.docx]

Additional measures and analyses for:

**Moral Conformity in a Digital World: Human and Nonhuman Agents as a Source of Social Pressure for Judgements of Moral Character**

Konrad Bocian^1*^, Lazaros Gonidis^2^, Jim A.C. Everett^3^

^1^ Department of Psychology in Sopot, SWPS University

^2^ School of Psychology, University of Sussex

^3^ School of Psychology, University of Kent

* Corresponding author

Email: [kbocian1@swps.edu.pl](mailto:kbocian1@swps.edu.pl)

**Study Materials**

Moral foundations vignettes (Clifford et al., 2015)

**Care Foundation**

Laugh

You see a girl laughing at another student forgetting her lines at a school play

Dinner

You see a man loudly telling his wife that the dinner she cooked tastes awful.

Spatula

You see a woman spanking her child with a spatula for getting bad grades in school.

Dog

You see someone leaving his dog outside in the rain after it dug in the trash.

**Fairness Foundation**

Player

You see a soccer player pretending to be seriously fouled by an opposing player.

Halloween

You see a girl taking all the Halloween candy from a bowl, leaving none for others.

Line

You see a boy skipping to the front of the line because his friend is an employee.

Hired

You see a woman getting hired only because her father is close friends with the boss.

**Authority Foundation**

Order

You see a girl ignoring her father's orders by taking the car after her curfew.

Teacher

You see a teaching assistant talking back to the teacher in front of the classroom.

**Loyalty Foundation**

General

You see a former UK General saying publicly he would never buy any UK product.

Cheerleader

You see a head cheerleader booing her high school's team during a homecoming game.

**Sanctity Foundation**

Toothbrush

You see a teenage male in a dorm bathroom secretly using a stranger's toothbrush.

Gay

You see a homosexual in a gay bar offering sex to anyone who buys him a drink.

Cousins

You see two first cousins getting married to each other in an elaborate wedding.

Vomits

You see a college student drinking until she vomits on herself and falls asleep.

**Social Norms**

Phone

You see a man making a phone call in a cinema and talking loudly.

Gift

You see a woman coming to a dinner without a gift for the hosts.

Hello

You see a woman answering a phone call with the word "goodbye" instead of "hello.

Desert

You see a woman eating dessert before her main entree arrives on the table.

**Additional analyses**

The prevalence of conformity can be read in Figures S1 (Study 1) and S2 (Study 2).

*Figure S1.* The prevalence of conformity in Study 1 (Human group pressure).

Conformed out of 20

*Figure S2.* The prevalence of conformity in Study 2 (Avatar group pressure).

| Table S1 |  |  |  |  |
| --- | --- | --- | --- | --- |
| *Results of the pairwise comparisons for the moral domains (other than care) used in the Study 1* | | | | |
| Foundation | Fairness | Social Norms | Sanctity | Loyalty |
|  |  |  |  |  |
| Authority | *t*(102) = -1.09, *p =* .277 | *t*(102) = 3.26, *p = .*002 | *t*(102) = 0.53, *p = .*600 | *t*(102) = 0.00, *p =* 1.00 |
| Loyalty | *t*(102) = 1.18, *p =* .239 | *t*(102) = 3.60, *p < .*001 | *t*(102) = 0.55, *p = .*584 | *-* |
| Sanctity | *t*(102) = 2.73, *p = .*041 | *t*(102) = 3.03, *p = .*003 | *-* | *-* |
| Social Norms | *t*(102) = 5.23, *p < .*001 | *-* | *-* | *-* |

| Table S2 |  |  |  |  |
| --- | --- | --- | --- | --- |
| *Results of the pairwise comparisons for the moral domains (other than care) used in the Study 2* | | | | |
| Foundation | Fairness | Social Norms | Sanctity | Loyalty |
|  |  |  |  |  |
| Authority | *t*(137) = 1.18, *p =* .238 | *t*(137) = 5.02, *p < .*001 | *t*(137) = 3.43, *p <.*001 | *t*(137) = 0.27, *p =* .786 |
| Loyalty | *t*(137) = 0.94, *p =* .349 | *t*(137) = 4.44, *p < .*001 | *t*(137) = 3.15, *p = .*002 | *-* |
| Sanctity | *t*(137) = 3.04, *p = .*003 | *t*(137) = 1.88, *p = .*062 | *-* | *-* |
| Social Norms | *t*(137) = 4.69, *p < .*001 | *-* | *-* | *-* |

**Study 1**

As can be seen in Table S1, additional pairwise comparisons for moral domains other than care showed that people conform less judging violations of sanctity (*M* = 0.46, *SD* = 0.31) and social norms (*M* = 0.36, *SD* = 0.26) than violations of fairness (*M* = 0.52, *SD* = 0.31). Moreover, people conform less frequently when targets transgressed social norms than sanctity (*M* = 0.46, *SD* = 0.31), authority (*M* = 0.48, *SD* = 0.40), and loyalty (*M* = 0.48, *SD* = 0.34).

**Study 2**

As can be seen in Table S2, additional pairwise comparisons for moral domains other than care showed that people conform less judging violations of social norms (*M* = 0.20, *SD* = 0.20) and sanctity norms (*M* = 0.25, *SD* = 0.26) than violations of fairness (*M* = 0.33, *SD* = 0.24), authority (*M* = 0.37, *SD* = 0.37), and loyalty (*M* = 0.36, *SD* = 0.33).

**Presence, realism, and IVE experience**

We asked participants about their experiences and feelings related to the IVE. According to a model of social influence within immersive virtual environments proposed by Blascovich et al. (2002), people should be more influenced by virtual agents if they believe that they exhibit: a) behavioural realism – the degree to which virtual objects appear to behave as they would in the physical world and b) agency – the extent to which avatars are perceived as a representation of a real person.

**Agency in the IVE** was measured with five items: “I felt that I was in the place/ environment that I saw”, “I felt that my interactions with other participants were natural”, ”The body I saw when I looked down, it belonged to someone else” (reversed-scored), “I had

the feeling that there were other people in the room”, “I had the feeling that the other participants understood my presence in the room”. Participants indicated to what extend they agree with each of the statements using a scale from 1 = *strongly disagree* to 7 = *strongly agree* (α = 0.64, *M* = 3.72, *SD* = 1.06).

**Realism in the IVE** was measured with five items: “I had the feeling that the other participants in the room were real people”, “I had the feeling that the other participants were acting like robots” (reversed-scored), “The answers given by the other participants sounded like nonhuman” (reversed-scored), “I felt that other participants answered like real people”, “I felt the presence of other people in the room”. Participants indicated to what extend they agree with each of the statements using a scale from 1 = *strongly disagree* to 7 = *strongly agree* (α = 0.77, *M* = 2.55, *SD* = 0.98).

**VR experience** was measured with a single item. Participants were asked to report their experience with VR on a scale from 1 = *No experience at all* to 5 = *a lot of experience* (*M* = 2.14, *SD* = 1.26).

We did not find any difference between human-controlled and AI-controlled avatars conditions in participants' feelings of presence in the IVE (*M* = 3.80, *SD* = 1.08 vs. *M* = 3.64, *SD* = 1.03, *t*(132) = 0.87, *p* = .385), realism in the IVE, (*M* = 2.64, *SD* = 1.01 vs. *M* = 2.46, *SD* = 0.94, *t*(132) = 1.07, *p* = .288), and VR experience (*M* = 2.13, *SD* = 1.15 vs. *M* = 2.16, *SD* = 1.38, *t*(132) = -0.13, *p* = .900).

**References**

Clifford, S., Iyengar, V., Cabeza, R., & Sinnott-Armstrong, W. (2015). Moral foundations vignettes: A standardized stimulus database of scenarios based on moral foundations theory. *Behavior Research Methods, 47*, 1178-1198. <https://doi.org/10.3758/s13428-014-0551-2>
